# Pelvic solitary fibrous tumors: clinical manifestations, surgical management and long-term outcomes

**DOI:** 10.3389/fonc.2026.1782133

**Published:** 2026-07-31

**Authors:** Xinji Yang, Lei Liu, Xiaofei Hou

**Affiliations:** Department of Urology, Peking University Third Hospital, Beijing, China

**Keywords:** adjuvant therapy, Doege-Potter syndrome, immunohistochemistry, pelvic solitary fibrous tumor, surgical resection

## Abstract

**Background:**

Pelvic solitary fibrous tumor (SFT) is a rare mesenchymal neoplasm with marked clinical and radiological heterogeneity, posing significant diagnostic challenges. Standardized management strategies remain lacking for special clinical scenarios, including paraneoplastic syndromes, synchronous primary malignancies, and pregnancy-associated incidental diagnosis.

**Methods:**

This single-center retrospective case series included 3 consecutive patients with histologically confirmed pelvic SFT who underwent surgical resection at Peking University Third Hospital between October 2018 and September 2025. Clinical, radiological, surgical, pathological, and long-term follow-up data were systematically collected, and a narrative literature review was conducted to refine clinical management.

**Results:**

The three patients presented with distinct rare phenotypes: a 17.5 cm giant tumor complicated by Doege-Potter syndrome, synchronous low-grade bladder urothelial carcinoma, and an asymptomatic tumor incidentally detected during twin pregnancy. All achieved R0 radical resection via individualized open, robot-assisted, or laparoscopic approaches, with no Clavien-Dindo grade ≥II perioperative complications. All tumors showed characteristic nuclear STAT6 positivity and CD34 expression, with uncommon MDM2/CDK4 and SMA/desmin co-expression. At median 36-month follow-up (3–84 months), no recurrence or metastasis was observed.

**Conclusions:**

Complete R0 resection is the cornerstone of pelvic SFT treatment. Individualized surgery is safe even for complex cases. STAT6 positivity is the diagnostic gold standard. High-risk patients may benefit from adjuvant radiotherapy, and lifelong follow-up is mandatory for late recurrence.

## 1 Introduction

Solitary fibrous tumor (SFT) is a rare mesenchymal neoplasm originally described in the pleura, but now recognized to occur in any soft tissue site throughout the body, including the pelvis (1–3). Pelvic SFT accounts for approximately 5–10% of all SFT cases and presents unique clinical challenges due to its deep anatomical location, potential for large size, and proximity to critical pelvic structures (4).

The clinical presentation of pelvic SFT is highly variable. Most patients are asymptomatic until the tumor reaches a large size, causing mass effect symptoms such as pelvic pain, urinary frequency, or constipation. Rarely, patients may present with paraneoplastic syndromes, most commonly Doege-Potter syndrome characterized by refractory hypoglycemia due to tumoral secretion of unprocessed insulin-like growth factor 2 (IGF-2) (5, 6). Concurrent malignancy with other pelvic tumors is extremely rare, with only isolated case reports in the literature (7).

Radiologically, pelvic SFT typically appears as a well-circumscribed, hypervascular mass, but these features overlap with many other pelvic neoplasms, including gastrointestinal stromal tumors (GIST), neurogenic tumors, and sarcomas (8). Definitive diagnosis requires histopathological examination, with nuclear STAT6 positivity resulting from the pathognomonic NAB2-STAT6 gene fusion being the gold standard (9).

Surgical resection with negative margins is the mainstay of treatment for localized pelvic SFT. However, the optimal surgical approach (open vs. minimally invasive) remains controversial, particularly for large or deeply located tumors (10). Furthermore, the role of adjuvant therapy for high-risk or recurrent disease is not well established, and standardized follow-up protocols are lacking (11).

Between October 2018 and September 2025, we treated three patients with pelvic SFT at our institution, presenting with three distinct and uncommon clinical scenarios. Herein, we report our experience with these cases, combined with a narrative review of the current literature, to provide insights into the diagnosis, surgical management, and long-term outcomes of this rare entity.

## 2 Materials and methods

### 2.1 Study design and patient population

This retrospective case series was conducted at Peking University Third Hospital, a high-volume tertiary urological center in China. The study protocol was reviewed and approved by the Biomedical Research Ethics Committee of Peking University Third Hospital. Patients were eligible for inclusion if they had histologically confirmed pelvic solitary fibrous tumor, underwent surgical resection at our institution between October 2018 and September 2025, and had complete clinical, radiological, and follow-up data available for analysis. Patients were excluded from the study if they presented with metastatic disease at initial diagnosis, had received prior treatment for pelvic SFT, or had incomplete clinical records that precluded comprehensive analysis.

### 2.2 Data collection

Relevant clinical data were retrospectively extracted from the electronic medical records of all included patients. The collected variables included demographic information such as age and gender, clinical presentation details including symptoms, symptom duration, and presence of paraneoplastic syndromes, preoperative evaluation results comprising laboratory tests, cross-sectional imaging findings (computed tomography and magnetic resonance imaging), and percutaneous biopsy results when performed. Additionally, detailed surgical data were recorded, including surgical approach, operative time, estimated intraoperative blood loss, transfusion requirements, and surgical margin status. Pathological findings such as tumor size, histological subtype, mitotic index, presence of necrosis, and complete immunohistochemical profile were also extracted, along with perioperative outcomes including postoperative complications graded according to the Clavien-Dindo classification system and length of hospital stay. Finally, follow-up data including surveillance imaging results, disease recurrence status, and overall survival were collected for all patients.

### 2.3 Follow-up protocol

All patients were followed postoperatively according to a standardized institutional surveillance protocol. Patients were scheduled for follow-up visits every 3 months during the first 2 years after surgery, every 6 months during years 3 through 5, and annually thereafter for life, given the well-documented risk of late recurrence in SFT which can occur up to 17 years after initial resection. At each follow-up visit, patients underwent a comprehensive physical examination, complete blood count, serum biochemistry panel, and contrast-enhanced computed tomography of the abdomen and pelvis to evaluate for local recurrence. Annual chest computed tomography was also performed to screen for distant metastatic disease. Any new or worsening clinical symptoms prompted immediate medical evaluation and additional diagnostic testing as clinically indicated.

## 3 Results

### 3.1 Patient characteristics

Three patients were included in this study, with a median age of 60 years (range 39–67 years). Two patients were male, and one was female. The clinical presentations were distinct: one patient had mass effect symptoms and Doege-Potter syndrome, one had hematuria due to concurrent bladder cancer, and one was asymptomatic with an incidental tumor detected during pregnancy. The baseline characteristics are summarized in **Table 1**.

**Table 1:** Baseline characteristics of the three patients.

Characteristic	Patient 1	Patient 2	Patient 3
Age (years)	60	67	39
Gender	Male	Male	Female
Clinical presentation	Dysuria, frequency, nocturnal hypoglycemia	Right flank pain, gross hematuria	Incidental finding on obstetric ultrasound
Tumor size (cm)	17.5 × 14.5 × 11.8	3.2 × 3.1	5.4 × 5.1 × 5.6
Surgical approach	Open	Robot-assisted laparoscopic	Laparoscopic
Operative time (min)	210	165	95
Estimated blood loss (mL)	800	150	100
Transfusion	2 units packed RBC	None	None
Length of stay (days)	10	4	5
Perioperative complications	None	None	None
Margin status	Negative (R0)	Negative (R0)	Negative (R0)
Follow-up duration (months)	3	18	84
Recurrence status	No evidence of disease	No evidence of disease	No evidence of disease

### 3.2 Clinical cases

#### 3.2.1 Case 1: giant SFT with Doege-Potter syndrome

A 60-year-old male presented with a 10-day history of dysuria, urinary frequency, and urgency. He also reported episodic nocturnal hypoglycemia characterized by restlessness, fatigue, and diaphoresis, which resolved with oral carbohydrate intake. Laboratory evaluation confirmed fasting hypoglycemia (2.9 mmol/L) with suppressed insulin and C-peptide levels, consistent with Doege-Potter syndrome.

**Figure 1:**
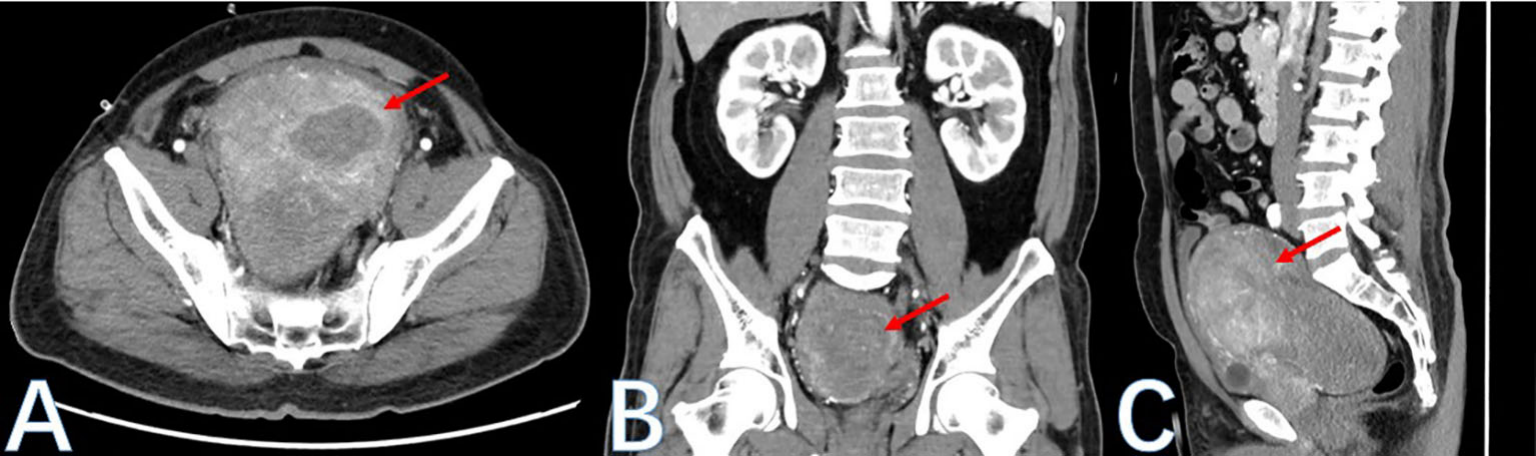
Computed tomography urography (CTU) images of the giant pelvic solitary fibrous tumor (SFT) in Case 1. **(A)** Axial contrast-enhanced CTU image showing a large heterogeneous soft-tissue mass measuring 17.5 × 14.5 × 11.8 cm with marked heterogeneous enhancement and a prominent superficial venous plexus. **(B)** Coronal contrast-enhanced CTU image demonstrating significant compression and displacement of the bladder, seminal vesicles, and rectum by the mass. **(C)** Sagittal contrast-enhanced CTU image confirming the well-circumscribed border of the giant tumor and its anatomical relationship with adjacent pelvic structures. Red arrow indicates the pelvic SFT.

CT urography (CTU) revealed a large, well-circumscribed pelvic mass measuring 17.5 cm in maximum diameter, causing marked compression of the bladder, seminal vesicles, and rectum (**Figure 1**). The mass showed heterogeneous enhancement on contrast imaging, with prominent feeding vessels. A CT-guided core needle biopsy confirmed the diagnosis of SFT.

The patient underwent open surgical resection via a midline laparotomy. Intraoperatively, the tumor was found to be well-encapsulated with a prominent superficial venous plexus. It was meticulously dissected from adjacent structures, and a wider deep margin was taken at the tumor base to ensure complete resection. The postoperative course was uncomplicated, and the patient was discharged on postoperative day 10. His hypoglycemic symptoms resolved completely immediately after surgery. At 3-month follow-up, surveillance CT showed no evidence of recurrence.

#### 3.2.2 Case 2: SFT concurrent with bladder cancer

**Figure 2:**
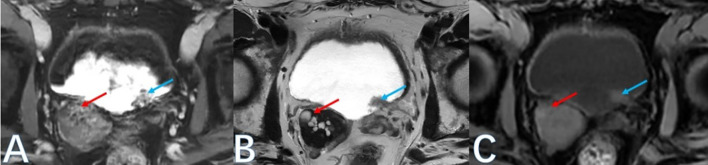
Magnetic resonance imaging (MRI) images of Case 2 demonstrating two distinct synchronous pelvic lesions. **(A)** Axial T1-weighted image (T1WI) showing a cauliflower-like soft-tissue lesion on the left posterior bladder wall with hypointense signal (blue arrow, ~12 × 8 mm) and a separate heterogeneous mass located in the right pelvis inseparable from the right seminal vesicle with hypointense signal (red arrow, ~3.2 × 3.1 cm). The prostate is enlarged with focal T1-hypointense areas. **(B)** Axial T2-weighted image (T2WI) revealing slightly hyperintense signal of the left posterior bladder wall lesion with ill-defined margins, and persistent hypointense signal of the right pelvic mass with indistinct borders. **(C)** Axial diffusion-weighted image (DWI, b=1000 s/mm²) showing marked hyperintensity (restricted diffusion) of the left posterior bladder wall lesion, while the right pelvic mass exhibits no definite diffusion restriction. The bladder lesion shows only mild enhancement after contrast administration, and the right pelvic mass shows heterogeneous enhancement on post-contrast sequences. No significant lymphadenopathy or pelvic fluid collection is identified. Red arrow indicates the pelvic solitary fibrous tumor located in the right pelvis; blue arrow indicates the low-grade papillary urothelial carcinoma located on the left posterior bladder wall.

A 67-year-old male presented with a 1-month history of right flank discomfort and gross hematuria. His serum PSA level was normal. MRI demonstrated two distinct lesions: a 1.2 cm cauliflower-like mass on the left posterior bladder wall and a 3.2 cm heterogeneous mass in the right pelvis inseparable from the right seminal vesicle (**Figure 2**). The bladder mass showed restricted diffusion on DWI, while the pelvic mass showed no diffusion restriction but marked heterogeneous enhancement.

The patient underwent a combined procedure: transurethral resection of the bladder tumor (TURBT) followed by robot-assisted laparoscopic resection of the pelvic mass. Cystoscopy confirmed a papillary tumor 0.5 cm lateral to the left ureteral orifice, which was completely resected. The pelvic mass was found to have a rich vascular pedicle originating from the internal iliac artery. After controlling the vascular supply, the tumor was excised completely with preservation of the seminal vesicle.

Final pathology confirmed low-grade papillary urothelial carcinoma of the bladder and SFT of the pelvic mass. The patient was discharged on postoperative day 4. At 18-month follow-up, he remained disease-free with no evidence of recurrence of either tumor.

#### 3.2.3 Case 3: asymptomatic SFT in a pregnant patient

A 39-year-old female with a twin pregnancy was found to have an incidental pelvic mass on routine obstetric ultrasound at 20 weeks gestation. She reported no urinary or constitutional symptoms. The mass was monitored throughout pregnancy with serial ultrasounds, which showed no significant growth. Elective resection was planned for the postpartum period.

**Figure 3:**
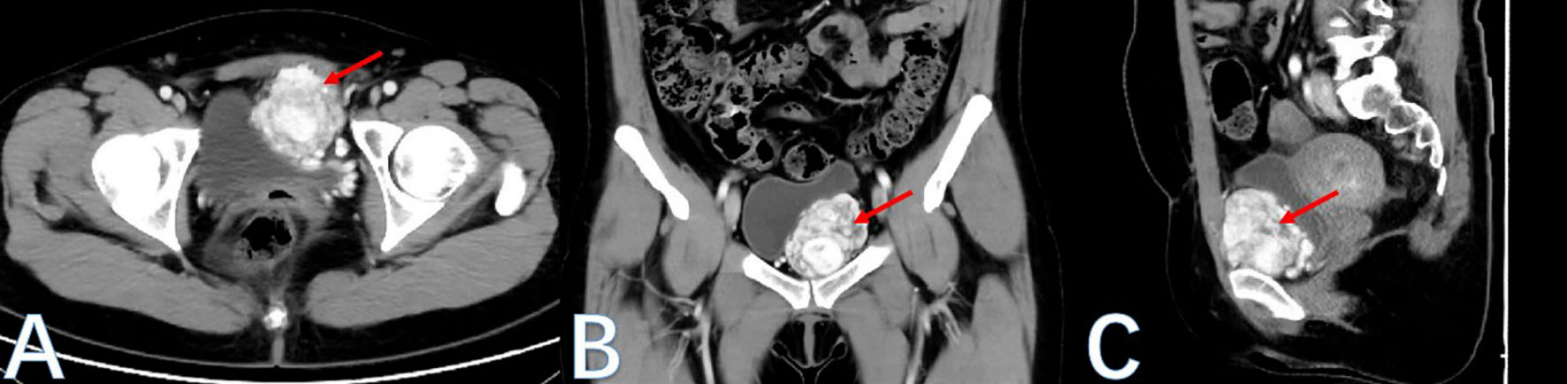
Computed tomography urography (CTU) images of the asymptomatic pelvic solitary fibrous tumor (SFT) in Case 3. **(A)** Axial contrast-enhanced CTU image showing a well-circumscribed, heterogeneous soft-tissue mass measuring 5.4 × 5.1 × 5.6 cm located anterosuperior to the bladder. **(B)** Coronal contrast-enhanced CTU image demonstrating marked heterogeneous enhancement during the arterial phase and multiple tortuous feeding vessels surrounding the lesion. **(C)** Sagittal contrast-enhanced CTU image confirming mild compression of the anterior bladder wall by the mass and clear interface with adjacent pelvic soft tissues. Red arrow indicates the pelvic SFT.

Four months after cesarean delivery, CTU revealed a well-circumscribed, hypervascular mass measuring 6 cm in maximum diameter, located anterosuperior to the bladder (**Figure 3**). Multiple tortuous feeding vessels were seen surrounding the lesion. The patient underwent laparoscopic resection via a preperitoneal approach. Intraoperatively, the tumor was found to be supplied by vessels from the retropubic space and left lateral bladder wall. It was dissected along its capsule and removed intact.

Histopathology confirmed SFT with no malignant features. The patient had an uneventful recovery and was discharged on postoperative day 5. At 7-year (84-month) follow-up, surveillance imaging showed no evidence of recurrence.

### 3.3 Pathological and immunohistochemical findings

All three tumors showed characteristic histological features of SFT: haphazardly arranged spindle cells, alternating cellular and hypocellular areas, and prominent staghorn-shaped blood vessels. No necrosis or increased mitotic activity (>4/10 HPF) was observed in any case.

Immunohistochemically, all tumors showed strong nuclear STAT6 positivity and diffuse or focal CD34 expression, confirming the diagnosis. Uncommon findings included weak MDM2 and CDK4 expression in Case 1, co-expression of smooth muscle actin (SMA) and desmin in Case 2, and BCL2 positivity in Case 3. The complete immunohistochemical profile is summarized in **Table 2**.

**Table 2:** Immunohistochemical profiles of the three patients.

Marker	Patient 1	Patient 2	Patient 3
STAT6	+	+	+
CD34	Focal +	+	+
Ki-67	<5%	<1%	~2%
SMA	Rare weak +	+	–
Desmin	–	+	NT
BCL2	NT	NT	+
MDM2	Weak +	–	–
CDK4	Weak +	–	–
PR	Individual cell +	–	–
S-100	–	–	–
CD117	–	–	–
DOG-1	–	–	–

NT, Not tested; +, Positive; -, Negative.

## 4 Discussion

### 4.1 Clinical and radiological features and differential diagnosis

Pelvic solitary fibrous tumor typically presents as a large, slow-growing mass, with most tumors exceeding 10 cm in diameter at the time of diagnosis (12). As demonstrated in our series, clinical manifestations are most commonly related to mass effect on adjacent pelvic structures, but approximately 10–15% of patients may present with paraneoplastic syndromes, the most frequent of which is Doege-Potter syndrome caused by tumoral secretion of unprocessed insulin-like growth factor 2 (5). Concurrent malignancy with other pelvic tumors is very rare, with only one reported case of SFT coexisting with bladder cancer in the English literature (13). Radiologically, pelvic SFT is characterized by a well-circumscribed mass with heterogeneous enhancement on both computed tomography and magnetic resonance imaging. On MRI, T1-weighted images typically show isointense signal relative to skeletal muscle, while T2-weighted images demonstrate heterogeneous high signal intensity interspersed with characteristic low-signal areas that correspond to dense collagen deposition within the tumor (14). Dynamic contrast-enhanced MRI reveals a “washout” enhancement pattern, which represents a valuable imaging feature for differentiating SFT from other pelvic neoplasms (15).

The differential diagnosis of pelvic SFT encompasses a broad spectrum of mesenchymal neoplasms with overlapping clinical and radiological features. Neurogenic tumors, including schwannomas and neurofibromas, represent the most common diagnostic challenge. Schwannomas typically exhibit higher T2 signal intensity than SFT, frequently contain non-enhancing cystic degenerative areas, and demonstrate a “persistent” or “plateau” enhancement pattern on DCE-MRI, while neurofibromas are often associated with neurofibromatosis and show diffuse strong S-100 protein positivity on immunohistochemistry (15). Desmoid fibromatosis is characterized by infiltrative growth with ill-defined borders, intermediate signal intensity on T2-weighted images, and relatively homogeneous enhancement, with nuclear β-catenin positivity representing a pathognomonic diagnostic feature (16). Well-differentiated liposarcomas contain macroscopic fat components that are readily identifiable on both CT and MRI, while dedifferentiated liposarcomas show rapid enhancement of non-fatty solid components; strong diffuse expression of MDM2 and CDK4 with corresponding gene amplification confirms the diagnosis of liposarcoma and distinguishes it from SFT (17). Gastrointestinal stromal tumors typically arise from the wall of the gastrointestinal tract and show characteristic immunoreactivity for CD117 and DOG-1, which are consistently negative in SFT4 (4).

### 4.2 Surgical management strategies

Complete en bloc surgical resection with negative surgical margins remains the cornerstone of treatment for localized pelvic SFT, as positive margins have been consistently associated with a significantly increased risk of local recurrence (6). Our series demonstrates the feasibility and safety of individualized surgical approaches tailored to tumor size, anatomical location, and patient-specific characteristics. Open surgery remains the gold standard for large (>10 cm) or locally invasive tumors, as it provides excellent exposure of the deep pelvic anatomy and allows for rapid control of intraoperative bleeding, which is of particular importance given the highly vascular nature of SFT (11). Robot-assisted laparoscopic surgery offers superior three-dimensional visualization and precise, tremor-free dissection, making it an ideal approach for deep pelvic tumors and combined procedures, as illustrated in our second case which required concurrent resection of a pelvic SFT and a bladder urothelial carcinoma (3). Laparoscopic surgery is well-suited for small to medium-sized, well-encapsulated tumors, particularly in young patients or those who would benefit from a minimally invasive approach, as demonstrated in our third postpartum patient¹². Preoperative transcatheter arterial embolization has been recommended for large (>10 cm) or highly vascular SFTs to reduce intraoperative blood loss and facilitate surgical resection (18). Although we did not perform preoperative embolization in any of our cases, it should be strongly considered preoperatively for tumors with prominent feeding vessels identified on cross-sectional imaging to improve surgical safety.

### 4.3 Pathological diagnosis and immunohistochemical pitfalls

Definitive diagnosis of SFT relies on histopathological examination combined with immunohistochemical analysis. The pathognomonic NAB2-STAT6 gene fusion results in strong nuclear STAT6 positivity, which has a sensitivity and specificity exceeding 95% for the diagnosis of SFT across all anatomical sites (5). CD34 is also a sensitive marker for SFT, but it is less specific and may be lost in high-grade or dedifferentiated tumors (19). As illustrated in our series, SFT may exhibit uncommon immunohistochemical features that can lead to diagnostic confusion, particularly when evaluating limited biopsy specimens. Weak focal expression of MDM2 and CDK4 may be observed in large SFTs, but unlike in liposarcoma, this expression is not associated with gene amplification and does not support a diagnosis of liposarcoma in the presence of STAT6 positivity (17). Focal expression of smooth muscle actin or desmin may indicate rare myoid differentiation in SFT, but this finding does not alter the diagnosis when accompanied by characteristic morphological features and STAT6 positivity (20). Progesterone receptor positivity, as observed in our first case, is extremely rare in pelvic SFT, and its clinical significance remains unknown at present.

### 4.4 Adjuvant therapy and risk stratification

The role of adjuvant therapy in the management of pelvic SFT remains controversial and requires further investigation. For low-risk patients defined by the Demicco risk model (tumor <10 cm, age <55 years, mitotic rate <4/10 high-power fields, and absence of necrosis) who undergo complete R0 resection, close observation is sufficient, as the risk of recurrence is low in this subgroup (21). However, for high-risk patients with positive surgical margins, large tumor size, high mitotic index, or tumor necrosis, adjuvant radiotherapy should be considered to reduce the risk of local recurrence (22). The recommended radiation dose is 50–66 Gy delivered in conventional fractions, and modern radiotherapy techniques such as volumetric modulated arc therapy or stereotactic body radiation therapy should be used to minimize toxicity to adjacent pelvic organs including the bladder, rectum, and small intestine (22).

For patients with metastatic or unresectable SFT, anti-angiogenic targeted therapy represents the first-line systemic treatment. Pazopanib has demonstrated the most promising clinical results, with the GEIS-32 trial reporting a 58% partial response rate and a 9.8-month median progression-free survival in patients with advanced SFT (23). Bevacizumab combined with temozolomide has also shown significant activity, particularly in patients with chemotherapy-resistant disease (23). Conventional cytotoxic chemotherapy has limited efficacy in non-dedifferentiated SFT and is generally reserved for dedifferentiated SFT, which is characteristically insensitive to anti-angiogenic therapy (24). Long-term surveillance must be individualized based on risk stratification, and lifelong follow-up is mandatory for all patients due to the well-documented risk of late recurrence, which can occur up to 17 years after initial resection (25). The recommended surveillance schedule includes imaging every 3–4 months for the first 2–3 years postoperatively (pelvic MRI or CT plus chest CT for distant metastasis screening), every 6 months during years 3–5, and annually thereafter. For high-risk patients, more frequent imaging may be warranted, and any new or worsening clinical symptoms should prompt immediate medical evaluation (21, 24).

### 4.5 Limitations

This study has several important limitations that should be acknowledged. First, it is a retrospective case series with a small sample size of only 3 patients, which limits the generalizability of our findings. Second, the follow-up duration is relatively short for two of the three patients, and longer follow-up is needed to fully assess the risk of late recurrence. Third, the literature review was narrative in nature rather than systematic, which may have introduced selection bias in the interpretation of previously published data.

## 5 Conclusion

Pelvic solitary fibrous tumor is a rare mesenchymal neoplasm that exhibits marked clinical and pathological heterogeneity, presenting with diverse clinical scenarios including Doege-Potter syndrome, concurrent bladder cancer, and incidental diagnosis during pregnancy. Complete surgical resection with negative margins remains the mainstay of treatment, and individualized surgical approaches including open, robot-assisted, and laparoscopic techniques can achieve excellent perioperative and long-term outcomes. Definitive diagnosis relies on nuclear STAT6 positivity on immunohistochemistry, and clinicians should be aware of uncommon immunohistochemical variants that may cause diagnostic confusion. High-risk patients may benefit from adjuvant radiotherapy, and all patients require lifelong follow-up due to the significant risk of late recurrence.

## Data Availability

The raw data supporting the conclusions of this article will be made available by the corresponding author upon reasonable request.
